# Function of microRNAs in the Osteogenic Differentiation and Therapeutic Application of Adipose-Derived Stem Cells (ASCs)

**DOI:** 10.3390/ijms18122597

**Published:** 2017-12-02

**Authors:** Walter M. Hodges, Frederick O’Brien, Sadanand Fulzele, Mark W. Hamrick

**Affiliations:** 1 Department of Cellular Biology & Anatomy, Medical College of Georgia, Augusta University, Augusta, GA 30912, USA; whodges@augusta.edu (W.M.H.); sfulzele@augusta.edu (S.F.); 2 Dwight D. Eisenhower Army Medical Center, Fort Gordon, Augusta, GA 30912, USA; fpobrien3@gmail.com

**Keywords:** TGFβ1, miR-17, miR-23a, miR-31, bone repair, BMP2

## Abstract

Traumatic wounds with segmental bone defects represent substantial reconstructive challenges. Autologous bone grafting is considered the gold standard for surgical treatment in many cases, but donor site morbidity and associated post-operative complications remain a concern. Advances in regenerative techniques utilizing mesenchymal stem cell populations from bone and adipose tissue have opened the door to improving bone repair in the limbs, spine, and craniofacial skeleton. The widespread availability, ease of extraction, and lack of immunogenicity have made adipose-derived stem cells (ASCs) particularly attractive as a stem cell source for regenerative strategies. Recently it has been shown that small, non-coding miRNAs are involved in the osteogenic differentiation of ASCs. Specifically, microRNAs such as miR-17, miR-23a, and miR-31 are expressed during the osteogenic differentiation of ASCs, and appear to play a role in inhibiting various steps in bone morphogenetic protein-2 (BMP2) mediated osteogenesis. Importantly, a number of microRNAs including miR-17 and miR-31 that act to attenuate the osteogenic differentiation of ASCs are themselves stimulated by transforming growth factor β-1 (TGFβ-1). In addition, transforming growth factor β-1 is also known to suppress the expression of microRNAs involved in myogenic differentiation. These data suggest that preconditioning strategies to reduce TGFβ-1 activity in ASCs may improve the therapeutic potential of ASCs for musculoskeletal application. Moreover, these findings support the isolation of ASCs from subcutaneous fat depots that tend to have low endogenous levels of TGFβ-1 expression.

## 1. Introduction

The defining feature of any stem cell population is the unique capability for self-renewal and uninhibited multi-potent lineage differentiation. Identification of mesenchymal stem cell (MSC) populations within various tissues has inspired numerous studies focused on the therapeutic application of MSCs. MSCs within adipose tissue were identified in 2001 via suction-assisted lipectomy, leading to a new focus directed towards better understanding their differentiation potential. This initial fibroblast-like population of cells was characterized as processed lipoaspirate (PLA) [1,2]. In addition to ease of acquisition, the PLA technique demonstrated significantly higher concentrations of MSCs compared to bone marrow aspirates [3]. Fraser et al. [4] demonstrated that stem cells in freshly isolated adipose stromal vascular fractions (SVF) can account for up to 3% of the aspirate composition, a 2500-fold increase compared to bone marrow-derived MSCs [4]. The combination of substantially higher yields per procedure, reduced immunogenicity, and the relative abundance of adipose tissue supports the growing therapeutic application of adipose-derived MSCs (ASCs) [1,2,3,4].

A role for adipose-derived stem cells in orthopaedic applications has been investigated in several areas: trauma, chronic wounds, tendon injuries, and degenerative conditions of tendons, muscles, and cartilage [5,6]. In many traumatic settings, particularly involving high-energy mechanisms of injury with associated soft tissue loss, fractures lack biological factors necessary for healing, and adjunctive treatments in tissue repair may facilitate improved clinical results [7,8]. Traumatic wounds with segmental osseous defects represent substantial reconstructive challenges [9,10,11]. Autologous bone grafting is considered the gold standard for surgical treatment of critical sized osseous defects in many cases, but donor site morbidity and associated post-operative complications remain a concern [12]. Recently, two separate groups demonstrated the clinical efficacy and safety of ASCs in humans with craniofacial hard-tissue injuries and proximal humerus fractures [13,14]. The ability of human ASCs to produce osteoid *in vivo* has previously been established [15], and techniques for adipose harvesting and isolation have undergone several generations of refinement [16]. Among other advantages, ASCs represent an abundant supply of stem cells with fewer donor site morbidities in contrast to corticocancellous autograft [12]. Autogenously grafted tissue also does not carry many of the safety risks, although reportedly low, associated with allograft material or commercially manufactured recombinant proteins such as BMP2 or BMP7 [17,18]. Although many aspects of ASCs in regenerative medicine require further investigation prior to clinical use in orthopaedic trauma, this technology holds enormous promise in this challenging area.

Understanding the signaling pathways, growth factors, and environmental milieu necessary for inducing pluripotent cells along an osteogenic lineage is essential for optimal utilization of this biological resource. MicroRNAs (miRNAs) were first discovered in *Caenorhabditis elegans* as short, noncoding, regulatory molecules approximately 22 nucleotides in length. Further work demonstrated that these small transcripts were more abundant than previously realized and regulated a wider scope of general, conserved cell processes [19]. Since that time considerable work has been performed to better characterize these small, non-coding RNAs and their unique regulatory functions. Importantly, miRNAs are now recognized to play key roles in mesenchymal stem cell quiescence, proliferation, and differentiation [20,21,22]. For example, miR-21 expression can repress Sprouty RTK signaling antagonist-2 SPRY2 and promote further osteogenic differentiation whereas miR-17 has been demonstrated to down-regulate the same process via inhibition of BMP2 [23,24]. This review highlights the capacity of miRNAs to alter cell populations in various adipose depots (e.g., subcutaneous vs. visceral white adipose tissue), and their potential to enhance the therapeutic application of ASCs for bone repair and regeneration.

## 2. Utilization of Adipose-Derived Stem Cells for Bone Repair

### 2.1. Tissue Sites for Harvesting ASCs

Although adipose tissue is widely available throughout the human body, the optimal source(s) for ASCs remains an area of ongoing study. Given the evidence demonstrating unique miRNA profiles for various types of tissues, it is important to determine the appropriate anatomical source and location of adipose tissue that might provide the optimal cell population based on their innate expression patterns [25,26]. Various studies have attempted to characterize the expression profiles of cells from different adipose depots [27,28,29]. Work by Klöting et al. [29] demonstrated that both visceral (omental) and subcutaneous adipose tissue share expression of over a hundred different miRNAs; however, 16 miRNAs were overexpressed in visceral tissue compared to subcutaneous fat. Importantly, two of these overexpressed miRNAs (miR-27a, and -29b) can facilitate osteogenic differentiation whereas another, miR-17, can suppress osteogenesis [23,30,31,32]. It is relevant to note here, however, that miR-27a and -29a were only elevated in fat depots from obese patients with type 2 diabetes mellitus, and not in fat depots from patients with normal glucose tolerance. Consistent with the idea that visceral fat may have greater osteogenic potential than subcutaneous fat, Peptan et al. [33] found that visceral adipose-derived stem cells harvested from rabbits possessed greater tendency towards osteogenic differentiation as measured by osteogenic markers. On the other hand, Tchkonia et al. [34] compared ASC replication in human subcutaneous, omental, and mesenteric cells, finding that subcutaneous ASCs demonstrated the highest regenerative capacity of the three populations. In a gender-based cell culture study comparing ASCs from superficial and deep subcutaneous adipose cells from both men and women, Aksu et al. [35] observed that in men cells harvested from the superficial layer were the most efficient in osteogenic differentiation compared to cells isolated from deeper layers. In contrast, cells from the superficial layer did not differ significantly in their osteogenic capacity from cells of the deeper layer among women, but cells from both layers in men showed greater osteogenic capacity than cells from either layer in women. While the surgical approach to obtaining adipose tissue is more rapid and less invasive for subcutaneous depots from the abdomen and thigh, the varying differentiation characteristics of tissue from different anatomical locations and from different genders should be considered during the development of clinical applications and merits additional study.

### 2.2. Donor Characteristics

In addition to aspiration site selection, ASC donor body weight and age can play a role in both the quality and quantity of acquired stem cells. The work of van Harmelen et al. [36] suggests that there is a consistent pool of ASCs relative to the number of adipocytes. Thus, an increase in overall body mass index (BMI) will correlate positively with a larger number of available stem cells. The differentiation capacity of the ASC population may, however, correlate negatively with overweight and obese classifications of BMI, potentially revealing a negative feedback mechanism via large numbers of hypertrophied adipocytes. This suggests that lower BMI for ASC donors is likely to be associated with cell populations having optimal osteogenic differentiation capacity. Surprisingly, increasing donor age may actually improve the osteogenic profile of ASCs. Specifically, bone marrow-derived mesenchymal stem cells demonstrate impaired proliferation, senescence, and chondrogenic potential with increasing age, whereas adipose-derived stem cells do not show these negative age-related effects [37]. In fact, while adipogenesis is known to increase in various tissues with age, the adipogenic potential of ASCs appears to be reduced with increasing age [37,38].

### 2.3. Harvesting and Isolating Adipose-Derived Stem Cells

**Figure 1:**
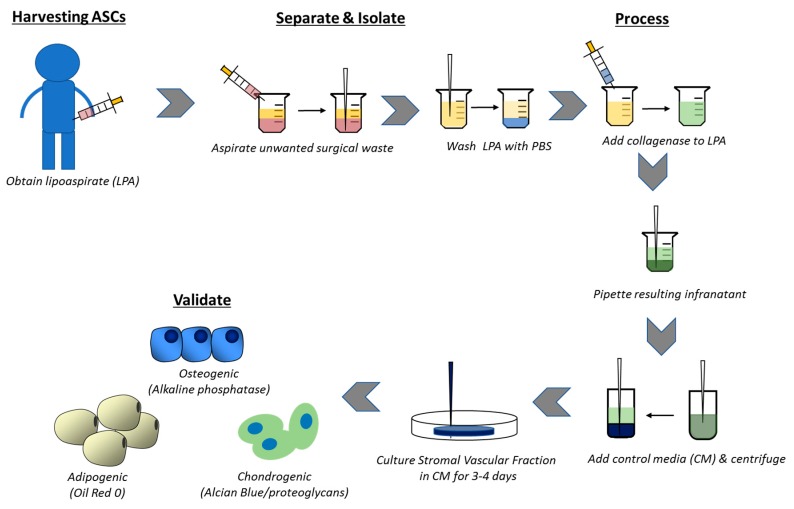
Schematic showing the processes and procedures for collection, isolation, and validation of adipose-derived mesenchymal stem cells (ASCs). The lipoaspirate (LPA) is frequently obtained from subcutaneous adipose tissue of the abdomen or thigh, and the miRNA expression pattern may differ among various adipose depots. Samples are washed and processed with collagenase to obtain a stromal vascular fracture that can be induced along osteogenic, adipogenic, or chondrogenic pathways.

Sequestration of ASCs from aspirated adipose tissue involves enzymatic digest to produce a more concentrated stromal vascular fracture (SVF). The SVF is defined by the presence of a heterozygous cell population including stem cells, macrophages, monocytes, endothelial cells, fibroblasts, smooth muscle cells, pericytes, as well as preadipocytes that have yet to develop any tissue specificity [39]. Stepwise culture and elimination of unwanted hematopoietic cell lines can produce a malleable stem cell product based on a variety of adherence specifications. Rodbell et al. [40] pioneered an early method for cell isolation from adipose tissue, and more recent approaches require collection of adipose tissue via needle biopsy or liposuction aspiration. Originally described by Zuk et al. [1], the manual isolation of ASCs from processed lipoaspirate cells (PLA) utilizes enzymatic digestion and differential centrifugation (Figure 1). The initial aspiration can be visualized as three distinct layers: a top oil layer from lysis of mature adipocytes, the desired middle layer containing raw adipose tissue, and a bottom layer of saline with cell populations including red blood cells. Both the top and bottom layers should be aspirated or decanted prior to further processing to ensure sample purity. The remaining middle layer is subjected to additional processing.

Once the middle adipose tissue layer has been isolated, it should then be washed with phosphate-buffered saline (PBS) that may be supplemented by antibiotics/antimycotics. Following further removal of debris, the sample should be added to a prepared sterile collagenase 1A solution within a filter unit. The digesting sample is incubated for approximately 30 min or longer depending on the presence of solid fat pieces. After digest, the sample is added to centrifugable tubes and 25 mL control medium (Dulbecco’s Modified Eagle’s Medium or DMEM containing heat inactivated fetal bovine serum and penicillin/streptomycin) added to each tube. Each vile is centrifuged and the supernatant aspirated, ensuring that the top oil layer and any floating adipocytes are aspirated with the supernatant. The SVF pellets are combined into a single tube along with 30 mL control medium and the centrifugation process is repeated and the supernatant removed. The SVF is purified by additional pipetting and resuspension along with multiple days of culturing the isolated cell population. Once this population is isolated, further phenotypic and functional characterization of the cells is performed. This typically involves flow cytometry to assess the cell surface CD antigen profile and then in vitro cell differentiation assays to evaluate differentiation potential [1,26,41]. Various differentiation protocols have been well outlined in the literature and should be referred to for additional cell processing.

### 2.4. Surface Markers for Validating Cell Populations

Following aspiration and processing, assessment of sample purity is essential for further work; however, a unique group of cell biomarkers for ASCs has yet to be universally identified. Expression of different genetic and cell surface markers varies among different cell variants within the ASC population. To further compound standardization issues, recent literature has demonstrated that post-aspiration processing can significantly impact surface phenotypic profiles with both gain and loss of particular biomarkers, and there is remaining disagreement over which markers adequately define the population. More recent work has demonstrated that isolated SVF aspirate will express hematopoietic identifiers such as CD11, CD14, CD34, CD45, and CD144. The presence of such markers can decrease or be lost entirely after multiple days in culture [42]. Conversely, further cell processing and serial experimental passages can both introduce and/or strengthen expression of various markers, including CD13, CD29, CD44, CD63, CD73, CD90, CD166 [43]. This distinction is important to separate freshly isolated aspirate (SVF) from laboratory processed samples.

With this in mind, the ASCs remain a relatively heterogeneous population [2]. As a mesenchymal lineage cell population, ADSCs express CD9, CD10, CD13, CD29, CD34 (+/−), CD44, CD49d, CD49e, CD54, CD55, CD63, CD73, CD90, CD105, CD146, CD166, and STRO-1 while hematopoietic markers like CD14 (lipopolysaccharide receptor), CD15, CD19 (B4), CD31 (endothelial cell adhesion molecule-1), CD34 (Mucosialin, +/−), CD45 (leukocyte common antigen), CD56 (NCAM), CD61 (integrin β3), CD62E (E-selectin), CD104 (integrin β4), CD106, and CD144 will be absent after additional processing [44,45] (Table 1). Stratifying the heterogeneous aspirate further, Li et al. [46] identified four important nonhematopoietic cell types within the SVF. These include preadipocytes (CD31(−)/CD34(+)/CD146(−)), pericytes (CD146(+)/CD31(−)/CD34(−)), and two CD31+ endothelial populations categorized as immature endothelial (CD31(+)/CD34(+)) and mature endothelial (CD31(+)/CD34(−)). Cells that were CD31+/CD34− (preadipocytes and immature endothelial) not only represented a significant majority of the extracted SVF (72.8%) but also demonstrated the greatest proliferation and highest adipogenic differentiation [3,46]. Optimizing extraction of the proper cell type will be essential to maximizing the regenerative benefits of therapeutic treatments.

There remains considerable debate concerning the viability of CD34 as a biomarker for the ASC population. As it stands, it is currently considered a potential marker than can identify an ASC subpopulation in situ and after a short time in culture [47]. Recent studies suggest that it might be a marker for both active and quiescent vascular stromal cells (VSC) within the capillaries and adventitia of larger blood vessels [48,49]. One such study was able to maintain a CD34+ phenotype for 6 weeks by suggesting that experimental conditions should mimic physiologic (cell-cell and cell-extracellular matrix) interactions as closely as possible [43]. Additional studies are needed to assess the role and function of CD34 as a marker for this population.

## 3. MicroRNAs mediating ASC osteogenic differentiation

**Figure 2:**
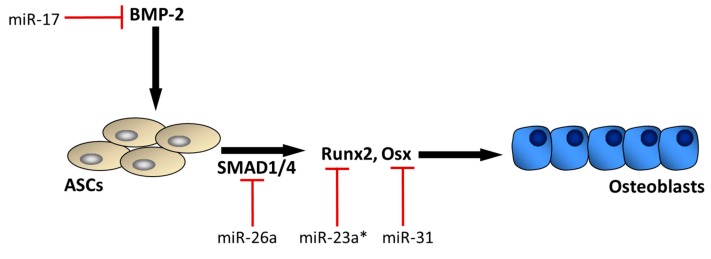
The BMP-2 signaling pathway plays an important role in the osteogenic differentiation of adipose-derived stem cells (ASCs). BMP-2 signaling induces downstream effectors SMAD 1 and SMAD 4, which in turn activate the key osteogenic transcription factors Runx2 and Osterix (Osx). Several miRNAs are expressed during this process that can attenuate this osteogenic pathway at various steps (red bars). * miR-23a interacts with Runx2, but there are numerous miRNAs targeting Runx2 in addition to miR-23a (Refs. [54,55]).

The osteogenic differentiation potential of human ASCs has been well established. Osteoblastic activity can be stimulated in ASCs following incubation with dexamethasone, β-glycerophosphate, l-glutamine, ascorbic acid, and/or vitamin D_3_, [26,50,51]. Cells cultured under these conditions subsequently express osteoblastic associated gene profiles including alkaline phosphatase, type I collagen, osteopontin, osteonectin, and Runx2 [2,51,52,53]. BMP signaling and its downstream transcription factors like ATF4, Osterix, & Runx2 are critical for the successful osteogenic differentiation of ASCs. Runx2 is perhaps most important in this respect, as it is the earliest cell specific transcriptional determinant known in the osteogenic lineage [30]. Runx2 is a member of the evolutionarily conserved family of DNA binding domain called the Runt domain through which Runx2 will interact with its nuclear constituents. Not surprisingly, miRNAs play a major role in mediating Runx2 activity during osteogenic differentiation [54] (Figure 2). A large number of miRNAs targeting Runx2 have been identified [54], and these miRNAs can interact with Runx2 in a complex manner. For example, while a number of these miRNAs such as miR-23a can suppress Runx2 activity, the expression of some of these miRNAs can in turn be altered by Runx2 itself [55].

Several miRNAs have been demonstrated to influence the multipotent differentiation potential of ASCs, but the network of miRNA interactions in this process has not been completely defined. miRNAs regulating the BMP-2 signaling pathway in ASCs appear to play a particularly important role in the osteogenic differentiation of these cells (Figure 2). For example, miR-17 is recognized as a potent inhibitor of BMP-2, and miR-17 can promote the adipogenic differentiation of ASCs by decreasing BMP-2 expression [23]. BMP-2 induces osteogenesis through the SMAD signaling pathway, which involves activation of both SMAD 1 and SMAD 4. The microRNA miR-26a can inhibit BMP signaling by blocking both SMAD 1 and SMAD 4 [56], and is observed to suppress the osteogenic differentiation of ASCs [57]. Likewise, miR-146a is also observed to suppress the osteogenic differentiation of ASCs by suppressing SMAD 4 [58]. SMAD 1 and SMAD 4 initiate transcription of the osteogenic factors Runx2 and Osterix (Osx). As noted above, numerous miRNAs such as miR-23a can target Runx2, and miR-31 is known to target Osx [59,60]. While several of these miRNAs play an anti-osteogenic role by suppressing the activity of various osteogenic genes, it is important to recognize that complex positive- and negative-feedback loops exist between these miRNAs and their targets [55]. In addition, several miRNAs have recently been identified that can enhance the osteogenic differentiation of ASCs. For example, miR-375 is upregulated during the osteogenic differentiation of ASCs and miR-375 overexpression enhances osteogenesis by targeting YAP 1 [61]. Endogenous miR-26a expression is also increased during the osteogenic differentiation of ASCs, and miR-26a overexpression promotes osteogenesis in ASCs by suppressing GSK3β [62]. Although there are several pro-osteogenic miRNAs whose functions are now well-established, miRNA profiling suggests that in general miRNAs tend to be elevated during the adipogenic differentiation of ASCs and suppressed during the osteogenic differentiation of ASCs [63].

## 4. Discussion

Traumatic injury to the craniofacial skeleton, limb, and spine presents a number of challenges for soft- and hard-tissue reconstruction. The relative abundance of adipose tissue as a stem cell source and the relative ease with which lipoaspirates can be obtained together support the clinical application of these autologous cells for regenerative medicine. There are, however, several areas that we believe merit further study in order to optimize this approach for bone repair. First, although the abdomen and inner thigh are commonly used as adipose sources for autologous grafting in plastic surgery procedures, the ideal tissue location for adipose tissue harvesting in orthopaedic applications has not been established. Subcutaneous and visceral adipose tissue can vary markedly in its gene expression profile [64], including variation in microRNA expression [29]. Moreover, subcutaneous fat can also differ in gene expression depending upon location, such as the abdominal or gluteal region [65]. An emerging area in stem cell therapy concerns the use of preconditioning strategies to optimize cell survival, proliferation, and differentiation. These strategies can involve treatment with growth factors, exposure to hypoxia, treatment with various pharmaceuticals, etc. as a means of optimizing cell performance [66]. In the case of ASCs one aspect of preconditioning to consider may be donor site location, since the intrinsic expression profile of the cell population is likely to vary among regions and may significantly impact the osteogenic capacity of the cell.

**Figure 3:**
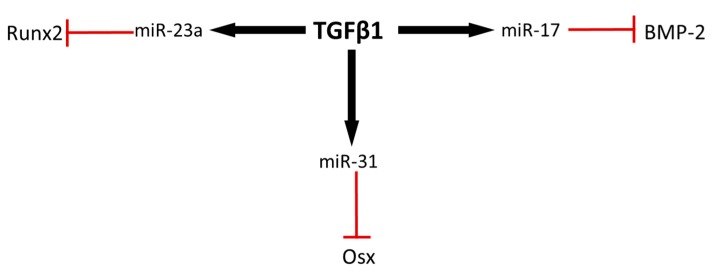
Transforming growth factor β 1 can induce expression of several anti-osteogenic miRNAs. miRNAs targeting osteogenic factors such as Osx, BMP-2, and Runx2 are stimulated by TGFβ1, potentially suppressing osteogenesis. Preconditioning strategies to attenuate TGFβ1 expression in ASCs may potentially enhance the osteogenic potential of these cells.

An additional strategy for optimizing the osteogenic potential of ASCs aside from donor site location might involve modification of the microRNAs that mediate osteogenic differentiation. We have highlighted several of these miRNAs that target various steps in BMP-mediated osteogenic differentiation (Figure 2). One strategy to increase the osteogenic potential of these cells could be to either expose the cells to antagomirs that inhibit these miRNAs, or to engineer cells that lack expression of these miRNAs. An alternative approach is to identify upstream mediators of these miRNAs and then target these upstream factors. A number of the miRNAs that can inhibit various steps in the BMP-2 signaling pathway appear to be mediated upstream by transforming growth factor β-1 (TGFβ1) (Figure 3). For example, miR-31 is induced by TGFβ1 in a variety of cell types [67,68,69], and miR-31 is a potent inhibitor of Osx. In addition, miR-17 is activated with exposure to TGFβ1 [70], and miR-17 directly inhibits BMP-2 activity. Finally, miR-23a, which is an important regulator of Runx2, is also induced by TGFβ1 [70]. Thus, preconditioning approaches that may inhibit TGFβ1 would likely improve the osteogenic potential of transplanted ASCs. This approach is consistent with profiling studies indicating that miRNAs enriched in the transforming growth factor-β signaling pathway are commonly downregulated during myogenic and osteogenic differentiation of ASCs [71]. TGFβ1 is a driver of fibrosis and while it is capable of inducing a number of miRNAs that suppress osteogenic differentiation it can also suppress microRNAs such as miR-206 that are important for myogenic differentiation [55]. These observations further support the notion that suppression of TGFβ1 activity in ADSCs may improve their utilization for musculoskeletal repair by enhancing not only bone regeneration but also muscle healing. Interestingly, TGFβ1 was previously shown to be downregulated in abdominal subcutaneous adipose tissue relative to subcutaneous adipose tissue from the gluteal region [65], perhaps suggesting that the use of abdominal subcutaneous ASCs may be preferable as a resource for musculoskeletal application.

## Figures and Tables

**Table 1:** Surface markers for cells of the stromal vascular fraction and for adipose-derived stem cells.

**Stromal Vascular Fraction**
**Positive:** CD11, CD13, CD14 (+/−), CD29, CD31, CD34(+/−), CD44, CD45 (+/−), CD49d, CD49e, CD55, CD63, CD73, CD90, CD105 (+/−), CD106 (+/−), CD117, CD144, CD146, CD166 (+/−), HLA-DR
**Negative:** CD11b, CD19, CD56, STRO-1
**Adipose-Derived Stem Cells**
**Positive:** CD9, CD10, CD13, CD29, CD44, CD49d, CD49e, CD54, CD55, CD63, CD73, CD90, CD105, CD144, CD146, CD166, HLA-ABC, CD34 (+/−), STRO-1
**Negative:** CD3, CD11b, CD14, CD19, CD31, CD34, CD45, CD56, CD62L, CD96L, CD117, HLA-DR
